# *N*-Alkylated Aminoacyl sulfamoyladenosines as Potential Inhibitors of Aminoacylation Reactions and Microcin C Analogues Containing D-Amino Acids

**DOI:** 10.1371/journal.pone.0079234

**Published:** 2013-11-04

**Authors:** Gaston H. Vondenhoff, Ksenia Pugach, Bharat Gadakh, Laurence Carlier, Jef Rozenski, Mathy Froeyen, Konstantin Severinov, Arthur Van Aerschot

**Affiliations:** 1 Laboratory of Medicinal Chemistry, Rega Institute for Medical Research, Leuven, Belgium; 2 Department of Molecular Biology and Biochemistry Waksman Institute, Rutgers, the State University, Piscataway, New Jersey, United States of America; 3 Institute of Gene Biology, Russian Academy of Sciences, Moscow, Russia; Bioinformatics Institute, Singapore

## Abstract

Microcin C analogues were recently envisaged as important compounds for the development of novel antibiotics. Two issues that may pose problems to these potential antibiotics are possible acquisition of resistance through acetylation and *in vivo* instability of the peptide chain. *N*-methylated aminoacyl sulfamoyladenosines were synthesized to investigate their potential as aminoacyl tRNA synthetase inhibitors and to establish whether these *N*-alkylated analogues would escape the natural inactivation mechanism *via* acetylation of the alpha amine. It was shown however, that these compounds are not able to effectively inhibit their respective aminoacyl tRNA synthetase. In addition, we showed that (D)-aspartyl-sulfamoyladenosine (i.e. with a (D)-configuration for the aspartyl moiety), is a potent inhibitor of aspartyl tRNA synthetase. However, we also showed that the inhibitory effect of (D)- aspartyl-sulfamoyladenosine is relatively short-lasting. Microcin C analogues with (D)-amino acids throughout from positions two to six proved inactive. They were shown to be resistant against metabolism by the different peptidases and therefore not able to release the active moiety. This observation could not be reversed by incorporation of (L)-amino acids at position six, showing that none of the available peptidases exhibit endopeptidase activity.

##  Introduction

**Figure 1:**
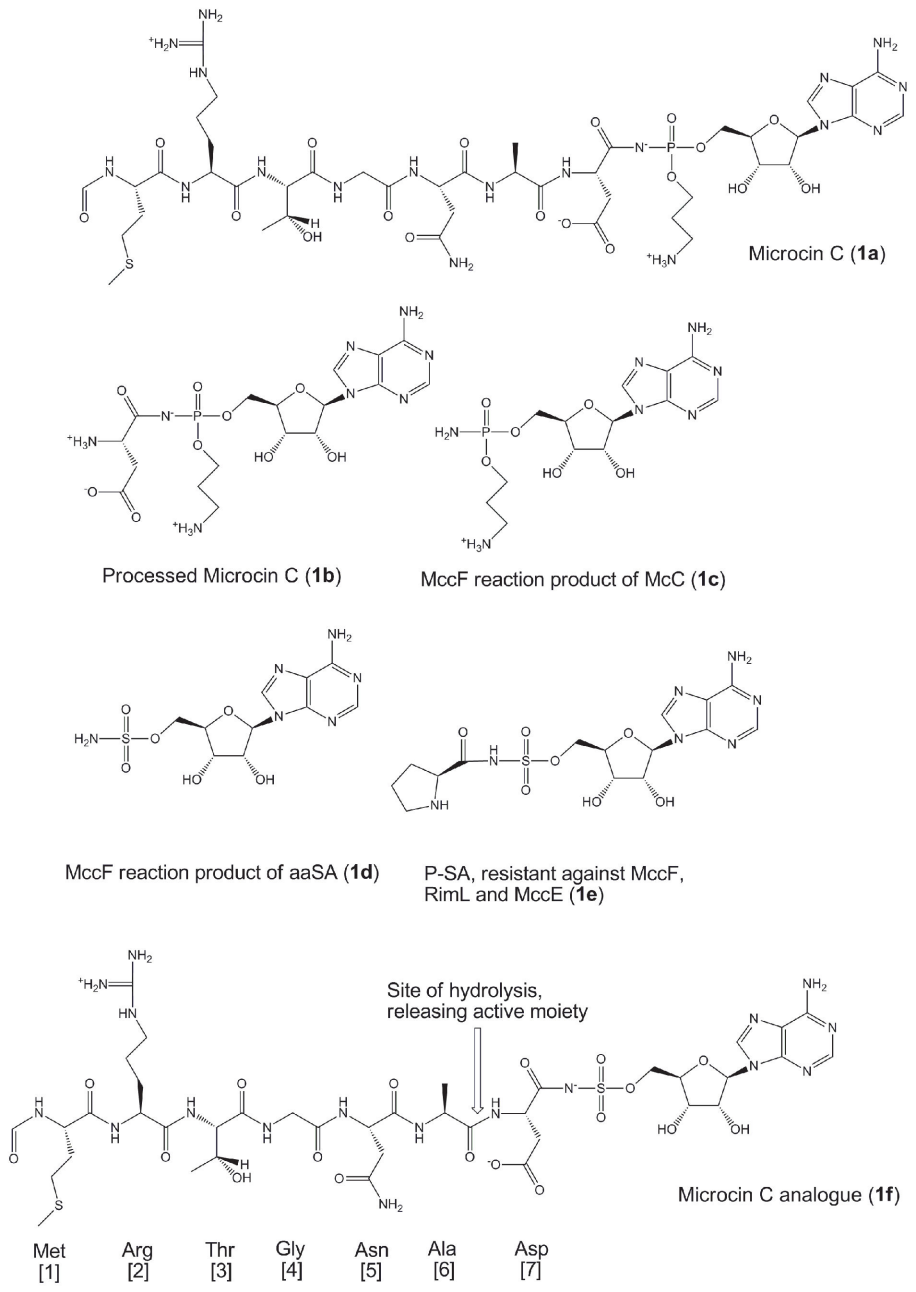
Structures for Microcin C (a), its metabolized form (1b), the reaction products of McC and its sulfamoylated analogues after metabolism by MccF (1c and 1d), Pro-SA (1e) which is fully resistant against MccE, RimL and MccF, and the earlier developed sulfamoylated McC analogue (1f).

Microcin C (McC) (**1a**, Figure 1) is a natural compound produced by Enterobacteriaceae. McC consists of a heptapeptide that is covalently linked through a phosphoramidate bond to adenosine, with in addition an aminopropyl moiety esterified to the phosphoramidate linker. In *Escherichia coli*, six genes of a plasmid-based *mcc* cluster (consisting of the *mccABCDE* operon and a separately transcribed *mccF* gene) determine the production, maturation, and secretion of McC, as well as provide self-immunity to the producing cell. The biosynthesis and the mode of action of McC have already been reviewed comprehensively elsewhere [1]. 

During recent years, the potency of this compound and its analogues, as well as its unravelled mode of action were firmly established [1-4]. However, to further pursue McC-like compounds as potential antibiotics, several concerns rise regarding i) foreseeable mechanisms of bacterial resistance and ii) *in vivo* stability of the peptide moiety.

One of the most obvious ways to adopt resistance comes from the self-producing cell. Once McC is assembled, it becomes prone to internal processing by different oligopeptidases, releasing processed McC, a nonhydrolysable aspartyl-adenylate analogue (1b) within the producing cell cytoplasm. Inevitable accumulation of processed McC in the producing cell should inhibit AspRS and lead to cessation of translation. However, McC producing cells carrying the entire *mcc* cluster continue to grow while producing McC. Indeed, the product of the *mccE* gene acetylates processed McC and converts it into a non-toxic compound [5]. Cells carrying the *mcc* operon with inactivated *mccE* grow slowly and apparently undergo self-poisoning by the McC they produce [5]. The MccE acetyltransferase is homologous to bacterial N-terminal acetyltransferases (NATs) of the Rim family. The *E. coli* genome encodes three Rim proteins, RimI, RimJ, and RimL, which acetylate ribosomal proteins S18, S5, and L12, respectively. The physiological functions of these NATs, and the significance of ribosomal proteins acetylation for cell physiology are not entirely clear. Our unpublished data indicate that RimL, but not RimI or RimJ, can detoxify processed McC and various other aminoacyl-nucleotides through acetylation of the alpha amine (T. Kazakov et al., in preparation) and thus contributes to the basic level of McC resistance. 

The *mccF* gene codes for a serine protease-like enzyme, and also provides resistance to both exogenous and endogenously produced McC. MccF detoxifies both intact and processed McC by cleaving the amide bond of the acyl phosphoramidate linkage, yielding product **1c** [6]. In addition it was shown that some aminoacyl sulfamoyladenosine (aaSA) analogues were likewise cleaved by MccF, releasing sulfamoyladenylate (**1d**), which was earlier shown to be a broad-spectrum antibacterial agent by itself [7]. However, while MccF can only effectively cleave aspartyl and glutamyl adenylates, RimL and MccE appear to acetylate and therefore inactivate a broad spectrum of aminoacyl adenylates with little specificity with respect to the nature of the aminoacyl moiety. Both findings suggest that bacterial resistance arising due to activation of a RimL/MccE type enzyme will be a more significant problem than resistance due to MccF.

We therefore set out to develop modified McC analogues that would be more resistant to the intrinsic self-immunity mechanisms of McC producing cells. To this end, two approaches were examined for their ability to prevent potential resistance to aaSAs and other aminoacyl tRNA synthetase (aaRS) inhibitors (such as isosters) of the aminoacyl-adenylates caused by acetylases such as MccE and RimL. In first place, we focused on modifying the aminoacyl moiety so that it would become resistant to acetylation. Secondly, the use of (D)-amino acids in aaSAs was explored to examine whether this would still be recognized by the corresponding aaRS and, subsequently, whether these could escape inactivation by MccE/RimL. 

It is well known that during evolution, cells developed specialized mechanisms to prevent the incorporation of (D)-amino acids in their proteins and ribosomally synthesized peptides. However, several (L)-aminoacyl-tRNA synthetases can transfer (D)-amino acids onto tRNA. This mis-esterification will however be corrected by (D)-aminoacyl-tRNA deacylases (DTD), which hydrolyze the ester bond [8]. Eukaryotes generally contain DTD1, while plants have DTD2 homologues [8]. Some bacteria, including most cyanobacteria lack genes encoding DTD1 homologues. It has also been reported that the editing site of ThrRS functions as a deacylase, removing non-cognate (D)-Thr [9]. In addition, several racemases, or in case of (D)-Glu a transaminase, can convert (D)-amino acids into (L)-amino acids. Alternatively, (D)-Ala, which is used for the formation of peptidoglycan in the bacterial cell wall, is produced from (L)-Ala by conversion *via* a racemase [10]. Despite the action of these corrective mechanisms, significant amounts of (D)-aminoacylated tRNA have been observed *in vitro* for (D)-Trp-tRNA^Trp^, (D)-Asp-tRNA^Asp^ [11], and (D)-Tyr-tRNA^Tyr^ [12,13]. 

Beside the resistance problem, from a pharmaceutical perspective the *in vivo* stability of the peptide moiety remains a concern. The introduction of (D)-amino acids in the peptide chain of McC at other positions than the C-terminal one is interesting as it has been shown in other cases to increase the plasma half-life of peptides [14]. Provided the bacterial cells can still release the active principle (*via* metabolism of the uptake promoting peptide sequence), this increase in plasma half-life would render the McC analogues much more interesting from a therapeutic perspective. Therefore, several analogues were created with (D)-amino acids to test this hypothesis and to establish to what extent the intracellular aminopeptidases can process peptides containing different combinations of (D)- and (L)- amino acids.

##  Results

### Design

5’-*O*-[*N*-[(L)-prolyl]-sulfamoyl]adenosine (Pro-SA, 1e) is not a substrate for either MccF or MccE [15]. Therefore, a McC analogue with proline at position seven would seem desirable, as it will overcome both resistance mechanisms. However, such compounds could not be synthesized, due to instability of these derivatives upon deprotection of the intermediates. This problem was also encountered earlier by Van de Vijver et al.[16], when studying dipeptidyl-sulfamoyladenosines containing proline at the C-terminal position. 

Nevertheless, the fact that Pro-SA (1e) was not inactivated by MccE, suggests that the presence of a secondary amine is sufficient to circumvent acetylation by this enzyme. Hence, we hypothesized that *N*-methylated aaSAs could also be resistant against acetylation by MccE. In contrast, the alpha amine of an amino acid usually serves as a hydrogen bond donor during esterification to the cognate tRNA as discussed by Nakama et al.[17] and as can be shown in many different structures (e.g. for AlaRS, a class II structure (Min Guo et al., [18]). We hypothesized however, that following *N*-methylation, this moiety still can be protonated and will be able to take part in ionic interactions or serve as a hydrogen bond donor. Examination of the *E.coli* AlaRS structures from Paul Schimmel's group (see previous ref: Min Guo et al.[18]) shows that the amine of all three co-crystallized ligands Gly-SA, Ala-SA and Ser-SA is forming only one hydrogen bond with Asp235 in the active site cavity. These authors studied the mistranslation of serine for alanine and also reported the amino acid binding pocket to be adjustable to different ligands. Visual inspection of the model already suggests that a single methyl group on the alpha amine could be easily accommodated. This is shown in Figure S1 for AlaRS with the modeled sarcosine analogue **3** sitting in the active site.

**Figure 2:**
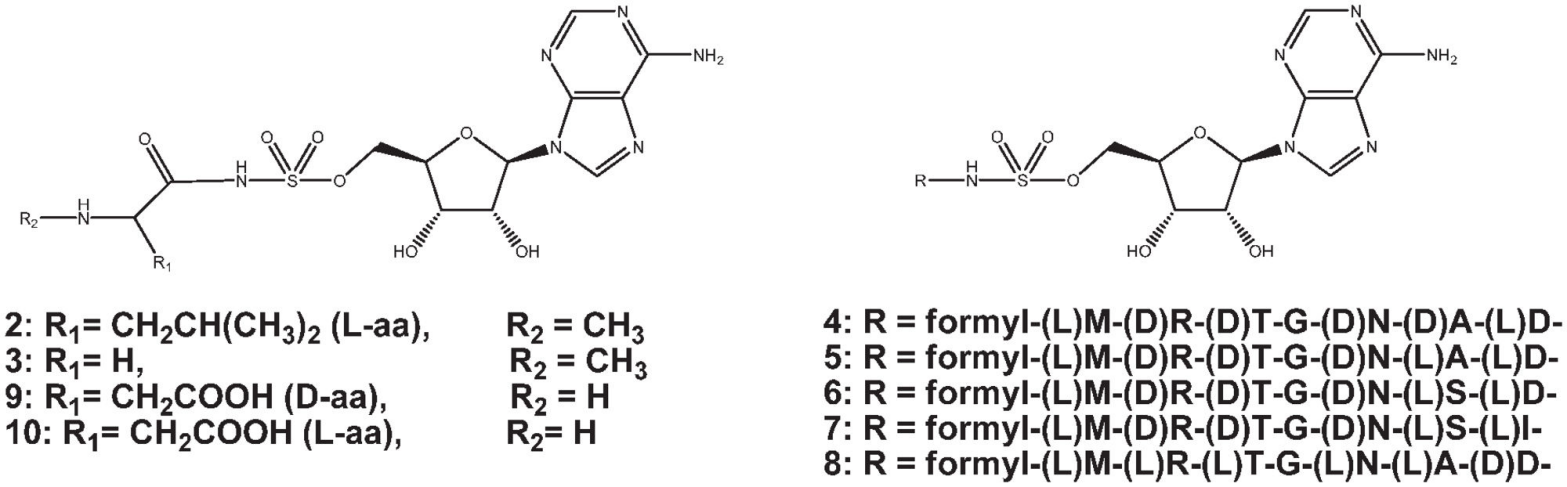
Structures for the *N*-methylated leucyl- (2) and glycyl-sulfamoyladenosine (3), the McC analogues containing (D)-amino acids (4-8), and the (D)-aspartyl- (9) and (L)-aspartyl-sulfamoyladenosine (10).

To further assess this idea, both 5’-*O*-[*N*-[(L)-*N*-methyl-leucyl]-sulfamoyl]adenosine (2) and sarcosyl-sulfamoyladenosine (3) (i.e. 5’-*O*-[*N*-[*N*-methyl-glycyl]-sulfamoyl]adenosine) (Figure 2) were synthesized and tested for their inhibitory properties. Compound **2** was selected for its straightforward synthesis, while compound **3** was selected for its rather small size, as we hypothesized that the latter might be active against either GlyRS and/or AlaRS (for all synthetic discussions we refer to the supplementary pages and the synthetic schemes (Figures S3 and S4)).

McC analogues comprising (D)-amino acids (Figure 2) were likewise synthesized to investigate if they would retain biological activity and at the same time would be resistant against the action of MccE and/or MccF. Hereto, McC analogues containing (D)-amino acids in the peptide tail (4-7) or as the C-terminal amino acid (8) were prepared. Compounds **4**-**8** were created to investigate the ability i) of cellular peptidases to process peptide bonds containing (D)-amino acids and ii) of the YejABEF transporter to recognize and facilitate entry of McC-like compounds with (D)-amino acids. In addition, we wanted to investigate whether compounds **8** and **9** ((D)-D-SA) would still inhibit the aminoacylation reaction and would have the potential to escape hydrolysis/acetylation by MccF and MccE respectively. 

### Biological Activity

**Figure 3:**
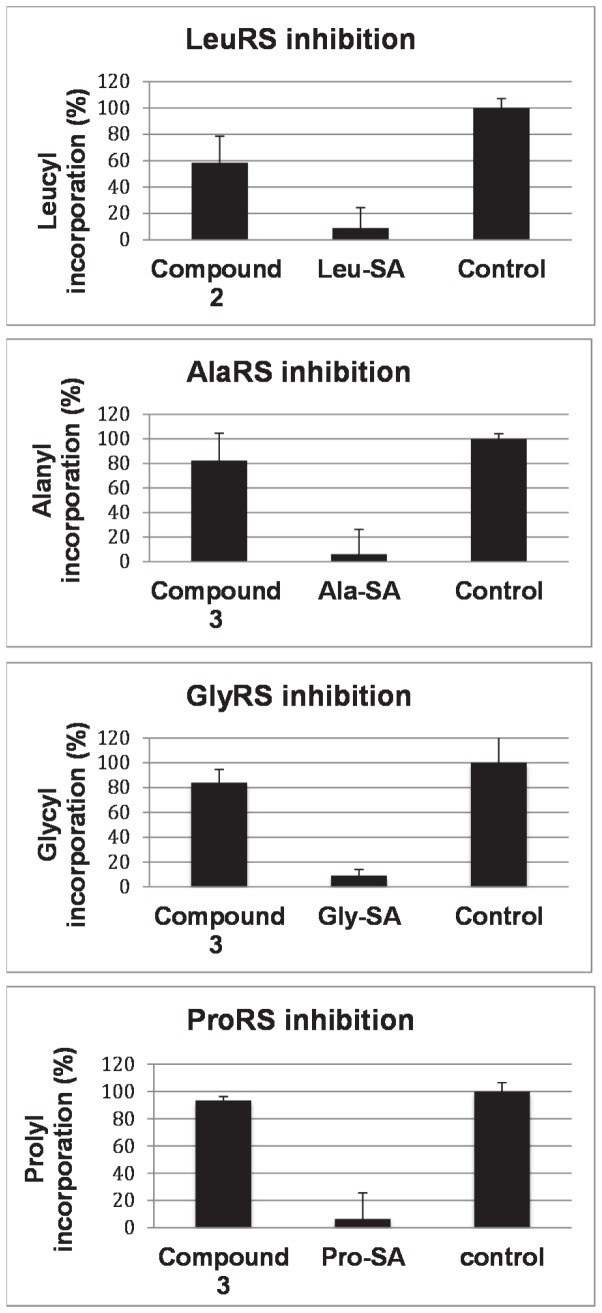
Lack of aminoacylation inhibition in presence of *N*-methylated leucyl- (2) and glycyl-sulfamoyladenosine (3) as potential inhibitors. Established aminoacyl-sulfamoyl-adenosines (Leu-SA, Gly-SA, Ala-SA and Pro-SA), that were previously shown to be potent inhibitors of their respective aaRSs, were used as positive controls. All experiments were performed in triplicate.

The growth inhibitory properties for all new compounds were determined by measuring the optical density reached by cultures of McC-sensitive *E. coli* in wells of microtiter plates in the presence of various concentrations of the respective inhibitors. As shown earlier, the intracellular target of McC analogs is determined by the C-terminal amino acid, which remains attached onto the sulfamoyladenosine following intracellular metabolism [19]. To facilitate activity evaluation and the mechanism of action studies of newly synthesized compounds, an *E. coli* tester strain Ara-Yej (BW39758) was used, where the *yejABEF* operon is under control of the arabinose-inducible *araBAD* promoter. In the presence of L-arabinose, higher amounts of Yej-transporters are displayed at the inner-membrane, making cells more sensitive to McC and related compounds [2]. In addition to the Ara-Yej strain, *E. coli* lacking *rim* genes (and therefore hypersensitive to McC) was used as a tester strain. The *N*-methylated aaSAs (2 and 3) proved however inactive in whole-cell activity assays with all cells tested (data not shown). In *in vitro* aminoacylation experiments, compound **3** was tested for its potential to inhibit ProRS, GlyRS and AlaRS and compound **2** was tested for its ability to inhibit LeuRS in a wild-type cell extract. Established inhibitors of these enzymes are the respective 5’-*O*-[*N*-[L-aminoacyl]-sulfamoyl]adenosines: Pro-SA, Gly-SA, Ala-SA and Leu-SA [2-4]. These compounds were therefore used as positive controls. Unfortunately, no activities comparable to the positive controls were observed at concentrations of 50 µM with either compound to either aaRS (Figure 3). 

**Figure 4:**
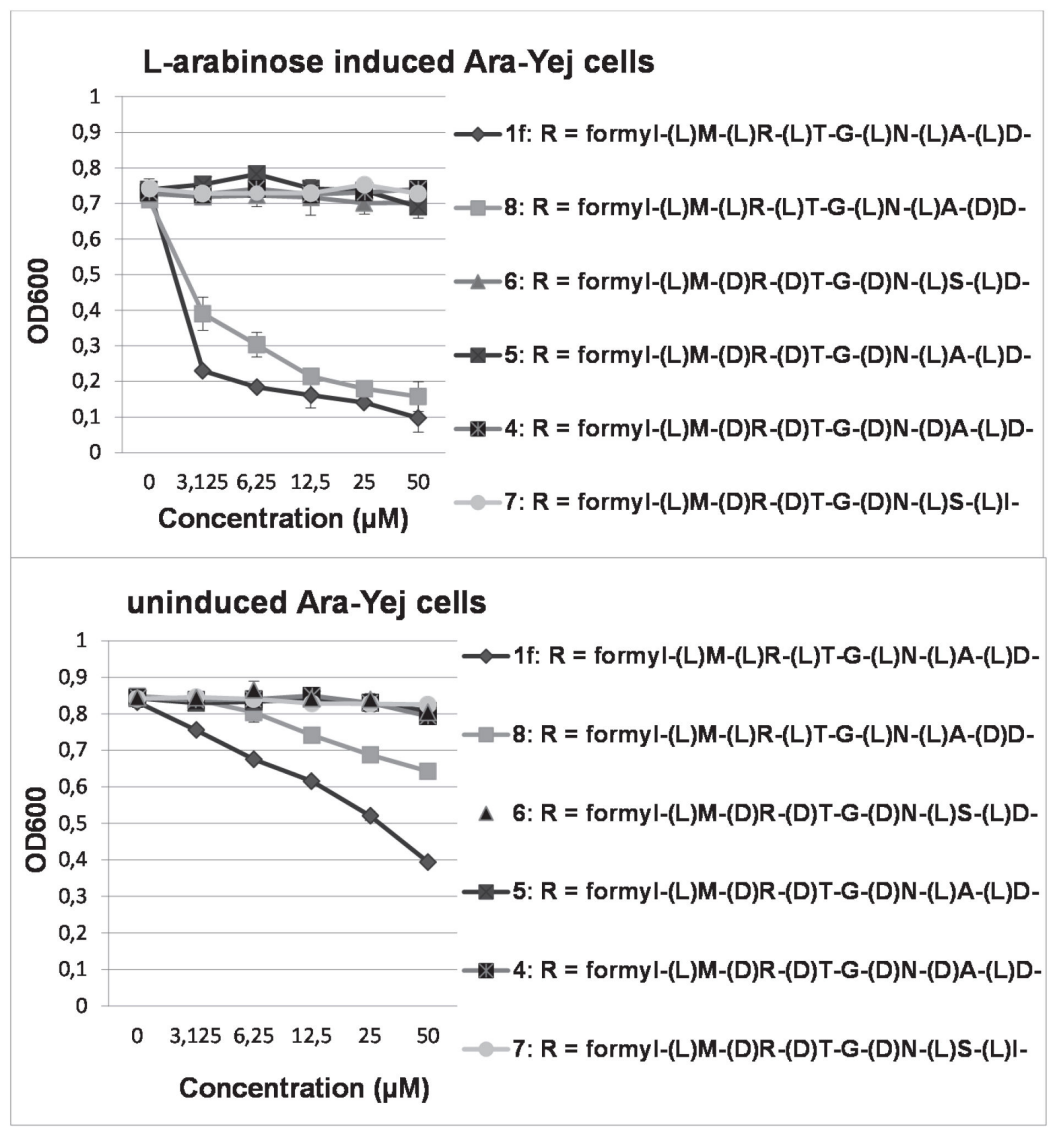
Inhibition profiles of the different McC analogues against (L)-arabinose induced Ara-Yej cells (upper panel) and against uninduced Ara-Yej cells, respectively. All experiments were performed in triplicate.

Of the newly synthesized McC analogues (4-8), only compound **8** showed significant activity in whole-cell activity screenings against Ara-Yej strain (Figure 4). The effect was more apparent upon the addition of arabinose to the growth medium (Figure 4, upper panel), indicating that the YejABEF transporter is involved in the intake. To confirm the Trojan horse mode of action, McC-resistant cells in which either an *yej* gene, or genes coding for peptidases A, B, and N had been disrupted were screened for activity with compound **1f** serving as a positive control. Both cells proved fully resistant to compound **8** (and to compound **1f**).

**Figure 5:**
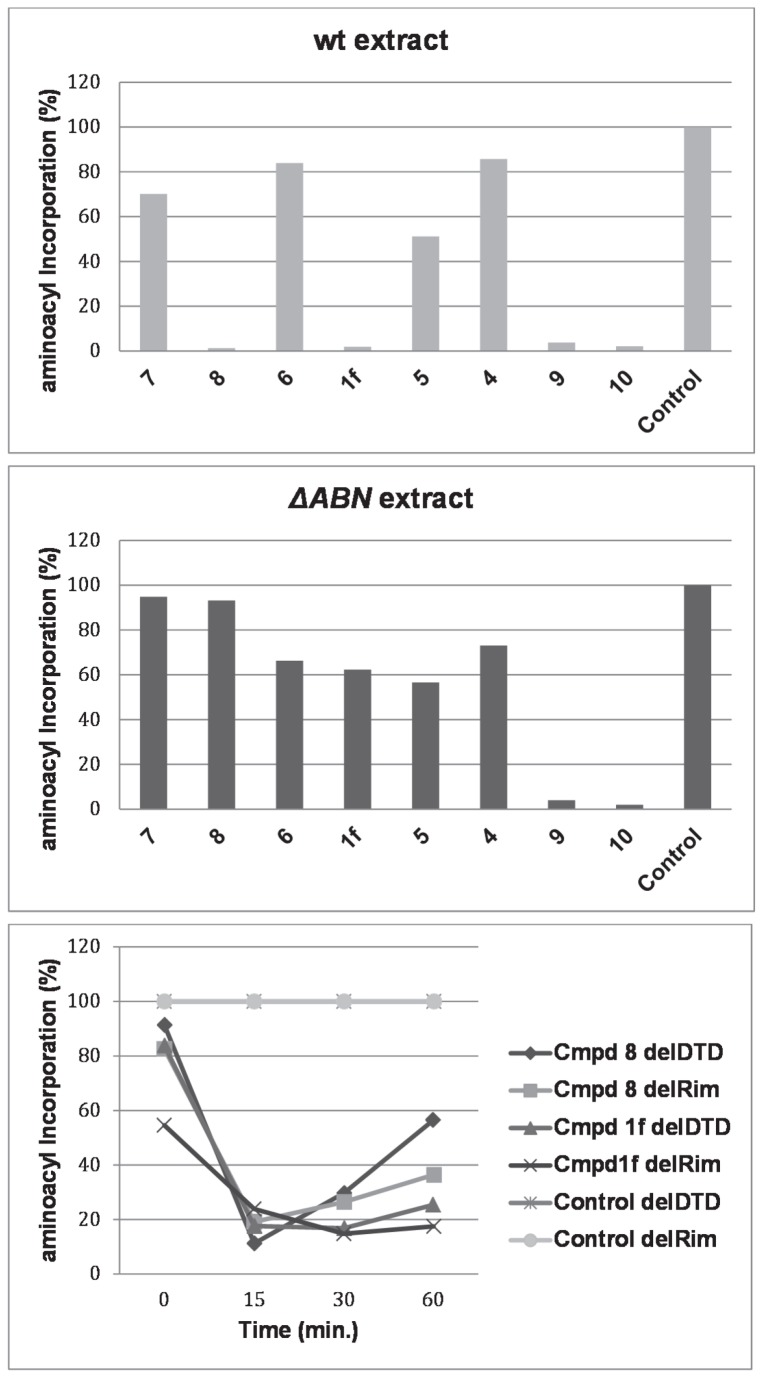
Aminoacylation experiments of the McC analogues containing (D)-amino acids at 50 µM. Upper panel: 30 min incubation with a cell extract deficient of RimL. Middle panel: 30 min incubation with a cell extract deficient of PepA, PepB and PepN. Lower panel: Comparison of two McC analogues targeting AspRS, compound **8** having a C-terminal D-configuration, compound **1f** having a C-terminal (L)-configuration. Incubation was done with two different cell extracts, lacking either DTD or RimL.

To further corroborate these results and to explain the absence of biological activity, *in vitro* studies were performed to test the ability of all compounds to inhibit tRNA aminoacylation in cell extracts prepared either from McC hypersensitive *E. coli* Δ*rimL* cells or from McC-resistant Δ*pepABN* cells. From Figure 5 it can be observed in agreement with the whole cell activity data, that only compound **8** behaved as tRNA aminoacylation inhibitor in extracts lacking Rim proteins, but had no effect in extracts lacking *N*-peptidases, confirming the requirement for at least one of these peptidases to release the active moiety. Since none of the McC analogues containing (D)-amino acids at positions two to six showed clear inhibition of AspRS or IleRS in extracts lacking RimL, relative to compounds **9** and **10** which do not require metabolism, we conclude that the presence of a (D)-amino acid within the McC peptide interferes with processing.

To investigate whether compound **8** would be more resistant than compound **1f** to MccF-catalyzed hydrolysis and/or MccE/RimL-catalyzed acetylation, and to investigate a possible role of DTD, a time-course of tRNA^Asp^ aminoacylation inhibition was followed in two different cell extracts, one prepared from cells lacking Rim proteins, and another from cells lacking DTD. From Figure 5 (lower panel) it can be observed that at time point zero, i.e., immediately after addition of the compounds, there was no inhibition since the compounds were still unmetabolized. However, following a time lapse sufficient for complete processing of wild-type McC, almost full inhibition of the AspRS-catalyzed reaction was observed with both compounds. Upon further incubation, the reaction recovered from inhibition for both compounds. For compound **8** this effect was more pronounced than for compound **1f**, especially in cell extracts prepared from cells lacking DTD proteins. Using the latter cell extracts, after 60 min, esterification of Asp-C^14^ to tRNA^Asp^ was reduced only 44% (compared to 89% inhibition or only 11% incorporation at 15 min). In case RimL was absent, both compounds largely remained inhibitory.

Compound **8** could still reach full activity, comparable to that of the (L)-D-SA analogue, in extracts prepared from cells lacking DTD, suggesting that both (D)-D-SA and (L)-D-SA are equally inhibitory to AspRS and therefore (D)-D-SA does not need to be isomerised by DTD to inhibit AspRS. This is in agreement with the observation of Soutourina et al. [11], that deacylases hydrolyze the ester bond of aminoacylated tRNA, rather than preventing esterification of (D)-amino acids to tRNA. One may also notice in Figure 5 (lower panel) that compound **8** suffers more from inactivation than compound **1f** in RimL deficient extracts. 

Compound **8** also showed whole cell activity against (L)-arabinose induced Ara-Yej cells, albeit to a lower extent than its (L)-analogue (Figure 4). None of the compounds **8** or **1f** showed activity against Δ*yej* or Δ*pepABN* cells (data not shown), confirming their Trojan horse mode of action.

##  Discussion

From the *in vitro* aminoacylation experiments with compounds **2** and **3** it can be concluded that these compounds lack inhibitory activity against their hypothesized targets (Figure 3). Although with proline, ProRS allows for a secondary amine within the binding site of the enzyme, Sar-SA (3) is not tolerated and does not show any major inhibitory binding affinity for this enzyme. In addition, GlyRS and AlaRS could not be targeted with this compound, showing that, although Sar-SA does not have a side chain like Gly-SA, this compound cannot establish sufficient binding within the active site of these enzymes. It thus can be concluded that the *N*-methyl group prevents inhibitory activity against GlyRS and AlaRS. Likewise, *N*-methylated Leu-SA (2) was found to be inactive against LeuRS. Again, it must be concluded that the *Nα*-methyl group in this compound also prevents inhibitory activity. This then suggests that the *Nα*-amine is an important recognition point inside the active site of the enzyme which cannot be modified. The recent results of Cusack et al. [20] published in the course of our work, and in which the aminoacylation and proofreading cycle of bacterial leucyl-tRNA synthetase was highlighted seem to sustain the latter hypothesis. In their crystal structure of the *E. coli* LeuRS in complex with the leucyl-adenylate analogue Leu-SA, the α-amine of the latter is hydrogen bonded to both the CO main chain of Leu41 and provides an ionic bond to the Asp80 side chain. *N*-methylation therefore not necessarily will prevent one of these H-bonds as the amine still can be protonated, but methylation is prohibited as it most probably would introduce unavoidable clashes (see supplementary pages and Figure S2 for further comments). In addition, since Sar-SA was not able to inhibit the aminoacylation reaction as catalyzed by ProRS it can be concluded that having a secondary alpha amine is not sufficient for recognition by ProRS, but that the five membered cyclic amine or at least a larger structure then the *N*-methylated glycine is required for recognition.

Compound **4** did not show whole cell activity either, and was also shown incapable of inhibiting AspRS *in vitro*. Since, the N-terminal amino acid is *N*-formyl-(L)-methionine, whereas amino acids at positions two to six are (D)-amino acids, it must be concluded that the peptidases are not capable of hydrolyzing (D)-amino acids and can therefore not liberate the active moiety. This is a somewhat expected result as it has frequently been shown that the introduction of (D)-amino acids can prevent the hydrolysis by peptidases [21].

Compounds **5**-**7** were especially designed to circumvent this problem; all three compounds contain L-amino acids at the sixth and seventh position, allowing endopeptidases to cleave between these two L-amino acids, resulting in release of the active moiety. Despite this modification, no activity could be observed. Also the modification in compound **6**, inspired by albomycin whereby pepN hydrolyzes the peptide bond between a serine and a modified amino acid carrying an acidic side chain [22], could not rescue the activity. From the *in vitro* aminoacylation experiments it can also be concluded that lack of whole cell activity is due to inability of the peptidases to metabolize peptides containing (D)-amino acids. Hence, this shows that the peptidases PepA, PepB and PepN, commonly known to be responsible for processing of McC and its analogues [23], only can cleave these compounds as exopeptidases (i.e. sequentially) and are not able to release the active moiety *via* endopeptidic cleavage (i.e. convergently). Whether or not incorporation of (D)-amino acids in the transport peptide part interferes with uptake of the McC derivatives by the YejABEF transporter is less relevant here, as *in vitro* tests already show lack of activity. Additional reduced uptake of the analogues **4-7** however cannot be excluded.

The observation that (D)-D-SA (9) and its McC derivative **8** can inhibit AspRS, shows that the peptidases can metabolize peptide bonds between two amino acids, whereby the C-terminal amino acid has the (D)-configuration (as in this case a (D)-Asp). This suggests that the peptidases involved in this reaction are only stereoselective for the N-terminal amino acid. 

Since it was already frequently observed that (D)-Asp can be esterified to tRNA, the finding that (D)-D-SA (9) can also inhibit AspRS can be considered being an expected result. This also shows that the absolute configuration of the amino acid is not required for recognition inside the active site of AspRS, and most probably the same holds for other aaRSs. This finding matches with the results of Thompson et al. [24] who concluded that there is only limited chiral specificity for L-Asp, leading to an esterification of (D)-Asp to tRNA^Asp^ with a rate of 1:4000 for (D)-Asp vs (L)-Asp.

These results are conflicting however with the views of Banik and Nandi [25-27] who studied the chiral discrimination by enzymes in protein synthesis *via* semi-empirical calculation methods. From their [24]theoretical studies they concluded that the network of electrostatic interactions between the incoming amino acid, ATP and the synthetase are highly unfavorable for incorporation of a (D)-amino acid. Not only in the aminoacylation step (relevant for our studies), but likewise in the peptide bond formation reactions, it would be virtually impossible to incorporate (D)-amino acids in protein structures. Our results however clearly demonstrate the *in vitro* inhibitory effects and hence recognition of our (D)-amino acid containing aminoacyl adenylate analogues.

Compared to compound **1f**, compound **8**, was more sensitive to inactivation by a mechanism that was not solely related to RimL. This can probably be ascribed to the intrinsic capacity of AspRS, favouring (L)-Asp over (D)-Asp. It is however unlikely that the editing site of AspRS is involved in this mechanism as it has often been shown to hydrolyze only non-cognate amino acids from tRNA. The inactivation of (D)-D-SA is also reflected in a lower whole cell activity for this compound, compared to its (L)-analogue (1f) (Figure 5). 

Four important observations were made in this study. First, we showed that *Nα*-methylation of Gly-SA and Leu-SA does not result in inhibition of the respective aaRSs and is therefore most probably not well tolerated in other aaRSs as well. *N*-methylation can therefore also not be considered as a preventive step against acetylases inactivating most aaSAs. Secondly, we showed that when one or more of the amino acids at positions two to six have a (D)-configuration, metabolism by the peptidases is abolished, thus preventing release of the active moiety and inhibition of the respective aaRS. In addition, it was shown that the peptidases primarily function as exopeptidases, as we did not observe significant inhibition of AspRS or IleRS with McC analogues containing an (L)-amino acid at the pre-C-terminal (or sixth) position. Third, if the C-terminal aspartic acid has a (D)-configuration and amino acids at positions one to six are in (L)-configuration, the active moiety was released, resulting in inhibition of AspRS. This shows that metabolism is independent of the C-terminal amino acid and only depends on the configuration of the N-terminal amino acid. Fourth, we have shown that (D)-D-SA is perfectly capable of inhibiting AspRS. However, this proved a relatively short lasting inhibition as over time the compound is inactivated by (predominantly) RimL. A less surprising finding, and therefore of lesser importance, is that DTD is not involved in the inhibitory activity of (D)-D-SA. 

## Materials and Methods

All synthetic procedures, materials used and analysis of the synthesized compounds can be found in the supplementary pages.

### Whole Cell Activity Determinations

The respective bacteria were grown overnight in Luria Broth (LB) medium and cultured again the following day in fresh LB medium or LB-medium containing 5 mM (L)-arabinose. Compounds were titrated in a 96-well plate using either LB-medium +/- 5 mM (L)-arabinose to dilute the compounds. To each well, 85 µL LB-medium +/- 5 mM (L)-arabinose was added to a total volume of 90 µL. Next, 10 µL of bacterial cell culture grown to an OD600 of 0.1 was added. The cultures were next placed into a Tecan Infinite M200® incubator and shaken at 37 °C, subsequently the OD600 was determined after 8 h. All experiments have been performed in triplicate.

Bacterial strains used for the evaluations: *E. coli* Ara-Yej (BW39758), expressing the *yejABEF* transporter upon (L)-arabinose induction; *E. coli* K-12 (BW28357), used as the wild type control; *E. coli ΔyejA*, lacking subunit A of the YejABEF transporter; and *E.coli ΔABN*, lacking all three peptidases *pepA*, *pepB and pepN*. ; *E. coli* Δdtd, lacking (D)-aminoacyl-tRNA deacylase

### Aminoacylation experiments

To assess the degree of inhibition of the aminoacylation reaction, *in vitro* tests were performed using the relevant S30 cell extracts.

#### Preparation of S30 cell extracts

Cells were grown in 50 mL LB-medium. After centrifuging at 3000 × g for 10 min. the supernatant was discarded and the pellet was resuspended in 40 mL buffer containing: Tris.HCl or Hepes.KOH (pH = 8.0) (20 mM), MgCl_2_ (10 mM), KCl (100 mM). The cell-suspension was centrifuged again at 4800 rpm. This procedure was repeated 2 times. The pellet was resuspended in 1 mL of the following buffer Tris.HCl or Hepes.KOH (pH = 8.0) (20 mM), MgCl2 (10 mM), KCl (100 mM), DTT (1 mM) and kept at 0 °C. Subsequently, the cells were sonicated for 10 sec. and left at 0 °C for 10 min. This procedure was repeated for 5-8 times. The lysate was centrifuged at 15000 g for 30 min at +4 °C. 

#### tRNA aminoacylation reaction

1 μL of solution containing inhibitor, 3 μL of *E. coli* S30 extracts was added. Next, 16 μL of the following aminoacylation mixture was added: Tris.HCl (30mM, pH 8.0), DTT (1 mM), bulk of *E. coli* tRNA (5 g/l), ATP (3 mM), KCl (30 mM), MgCl_2_ (8 mM), and the specified, radio labeled amino acid (40 μM). The reaction products were precipitated in cold 10% TCA on Whatman 3MM papers, 5 min. after the aminoacylation mixture was added. The aminoacylation reaction was carried out at room temperature. Depending on whether or not processing was needed, variable time intervals were included between the addition of the cell-extract and the addition of the aminoacylation mixture. After thorough washing with cold 10% TCA, the papers were washed twice with acetone and dried on a heating plate. Following the addition of scintillation liquid, the amount of radioactivity was determined in a scintillation counter.

## Supporting Information

Figure S1 **AlaRS starting structure from 3hxu.pdb with sarcosine substituting for alanine in the acive site.**
(TIFF)

Figure S2 **LeuRS starting structure from 4aq71.pdb with a modelled *N*-methylleucine derivative in the acive site.** Top: introducing the additional methyl moiety on the inhibitor (magenta) gives clashes with the nucleotide 76b (shown in green). ionic/H-bond distances are indicated to asp80, to leu41 and to nucleotide 76b.O2'. Bottom: the tRNA is removed from the model and CA-CB torsion angle of asp80 is adjusted slightly to accommodate the methyl moiety without clashes.
(TIFF)

Figure S3 **Synthetic scheme for synthesis of the protected *N*-methylated buiding blocks.** Upper part: Synthesis of *N*-methylated and succinimide activated Boc-Leu-OH. *i*) NaH, MeI in THF, 0 °C, 30 min. ii) EDCI.HCl, HOSu, DIPEA in DMF, rt, 16 h. Lower part: Synthesis of Cbz-protected and succinimide activated sarcosine. *i*) benzyloxycarbonyloxyl succinimide, NaHCO_3_ in H_2_O/dioxane, 0°C to rt, 7 h. ii) HOSu and EDCI.HCl in DMF, rt, 16 h.
(TIFF)

Figure S4 **General scheme affording the various McC analogues.** *i*) *N*-α-CBZ-(L)-aminoacyl-(*t*Bu or Boc)-succinimide, DBU in DMF, 6h, rt. ii) for R_2_ = Z-group, H_2_, Pd/C in MeOH, 3h, rt. iii) for R_2_ = Boc-group, TFA/H_2_O (5:2), 4h, 0 °C to rt. iv) Et_3_N.3HF in THF, 16h, rt. *v*) the respective protected peptide (1eq.), HOBt (4 eq.), DIC (4eq.) and DIEA (2 eq.) in DMF, 16h, rt. vi) TFA/thioanisole/H_2_O (90/2.5/7.5), 2h, rt.
(TIFF)
