# Supplementary figures and images for: N-Alkylated Aminoacyl sulfamoyladenosines as Potential Inhibitors of Aminoacylation Reactions and Microcin C Analogues Containing D-Amino Acids
Source: PLoS One. 2013 Nov 4;8(11):e79234. doi: 10.1371/journal.pone.0079234 (PMC3817062; doi:10.1371/journal.pone.0079234)

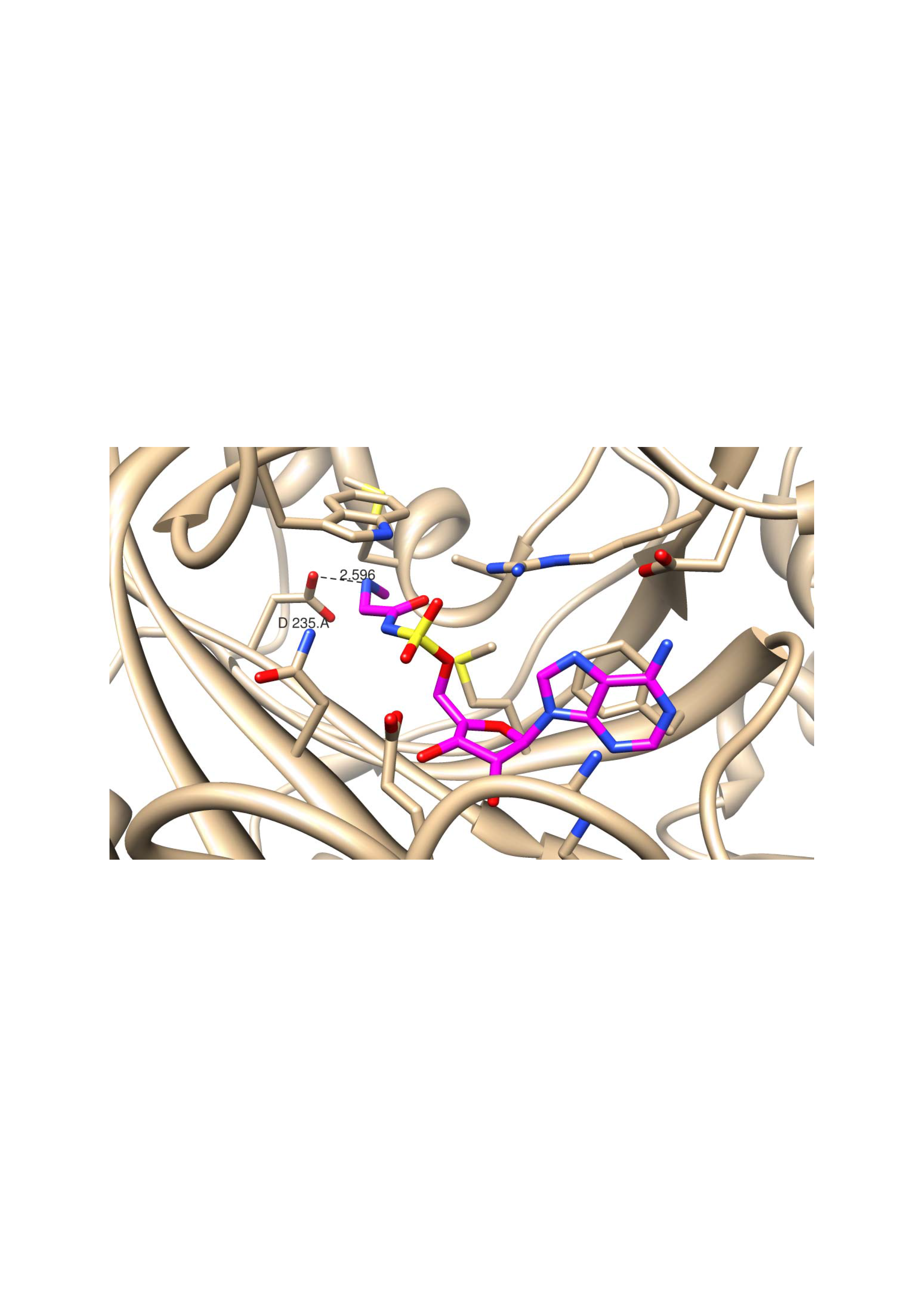

Supplement: Figure S1 — AlaRS starting structure from 3hxu.pdb with sarcosine substituting for alanine in the acive site. (TIFF) [file pone.0079234.s001.tiff]

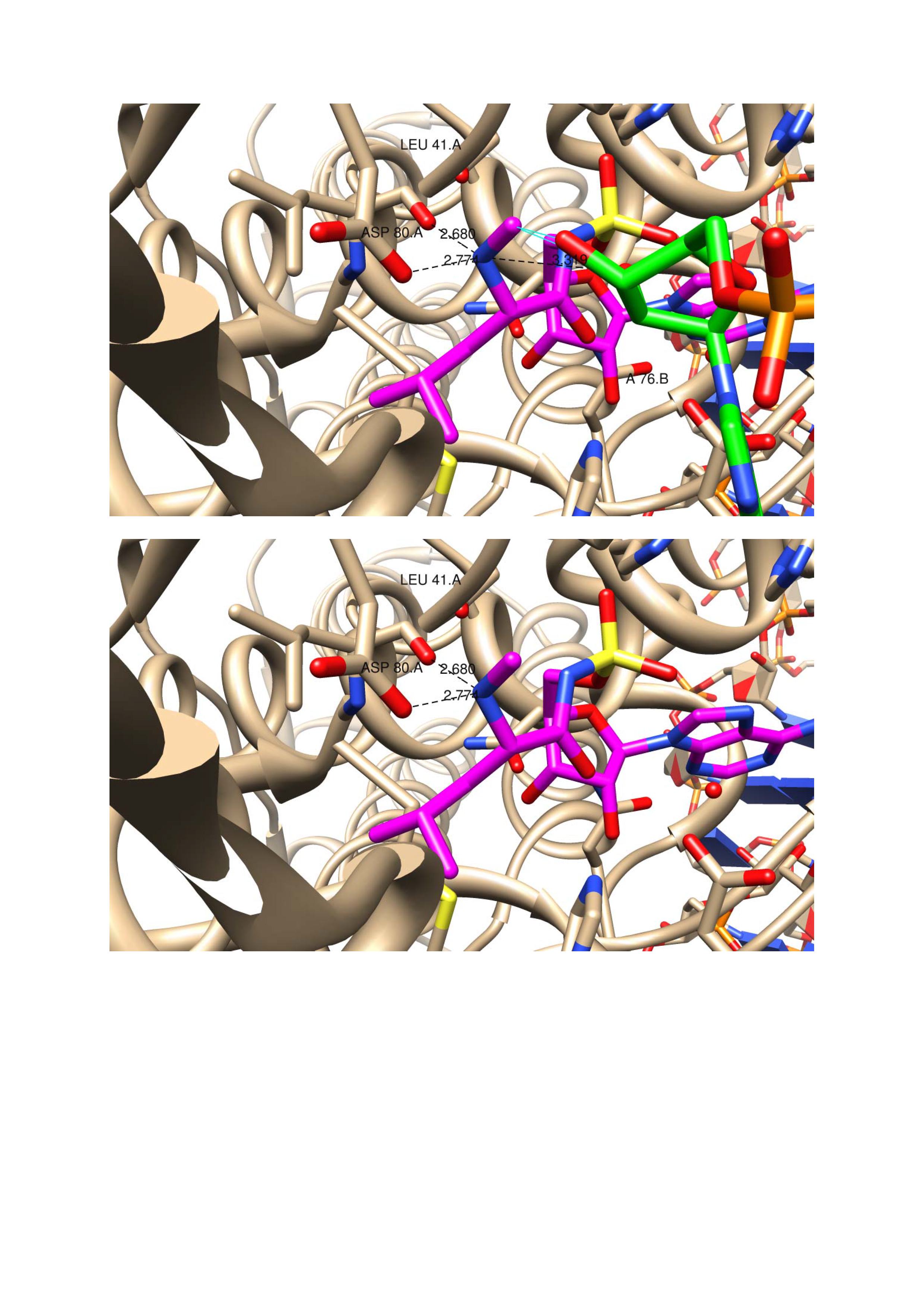

Supplement: Figure S2 — LeuRS starting structure from 4aq71.pdb with a modelled N-methylleucine derivative in the acive site. Top: introducing the additional methyl moiety on the inhibitor (magenta) gives clashes with the nucleotide 76b (shown in green). ionic/H-bond distances are indicated to asp80, to leu41 and to nucleotide 76b.O2'. Bottom: the tRNA is removed from the model and CA-CB torsion angle of asp80 is adjusted slightly to accommodate the methyl moiety without clashes. (TIFF) [file pone.0079234.s002.tiff]

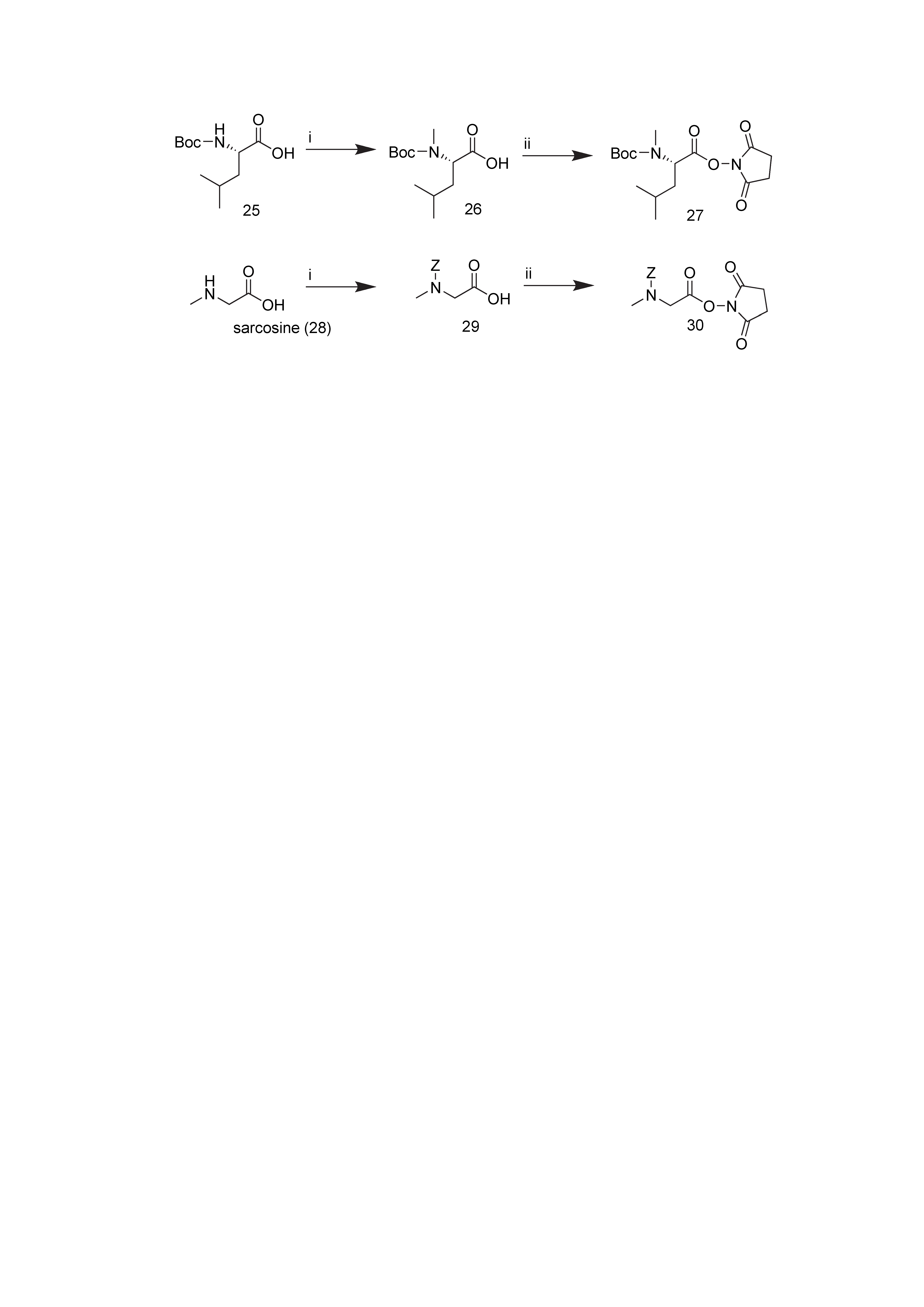

Supplement: Figure S3 — Synthetic scheme for synthesis of the protected N-methylated buiding blocks. Upper part: Synthesis of N-methylated and succinimide activated Boc-Leu-OH. i) NaH, MeI in THF, 0 °C, 30 min. ii) EDCI.HCl, HOSu, DIPEA in DMF, rt, 16 h. Lower part: Synthesis of Cbz-protected and succinimide activated sarcosine. i) benzyloxycarbonyloxyl succinimide, NaHCO3 in H2O/dioxane, 0°C to rt, 7 h. ii) HOSu and EDCI.HCl in DMF, rt, 16 h. (TIFF) [file pone.0079234.s003.tiff]

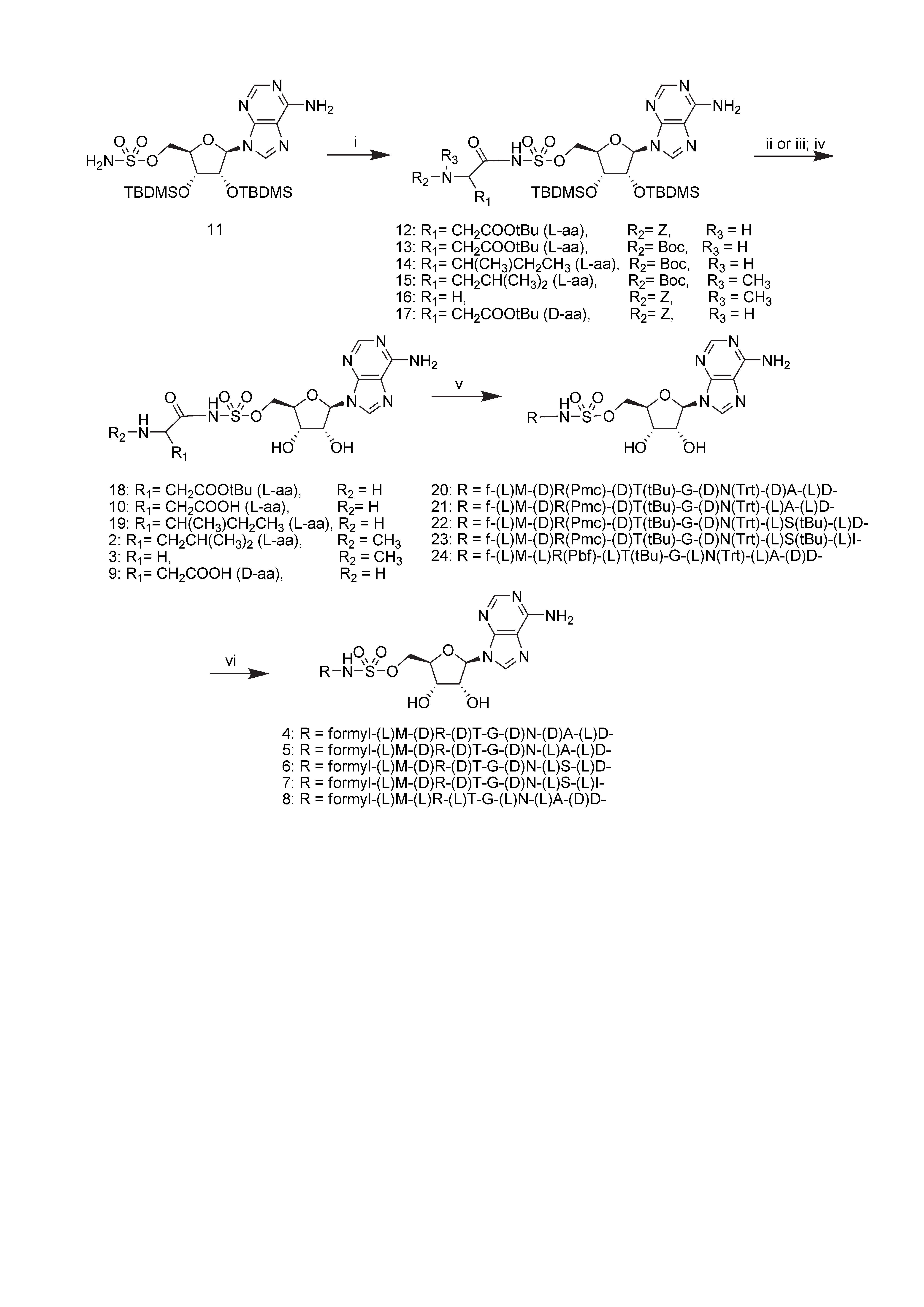

Supplement: Figure S4 — General scheme affording the various McC analogues. i) N-α-CBZ-(L)-aminoacyl-(tBu or Boc)-succinimide, DBU in DMF, 6h, rt. ii) for R2 = Z-group, H2, Pd/C in MeOH, 3h, rt. iii) for R2 = Boc-group, TFA/H2O (5:2), 4h, 0 °C to rt. iv) Et3N.3HF in THF, 16h, rt. v) the respective protected peptide (1eq.), HOBt (4 eq.), DIC (4eq.) and DIEA (2 eq.) in DMF, 16h, rt. vi) TFA/thioanisole/H2O (90/2.5/7.5), 2h, rt. (TIFF) [file pone.0079234.s004.tiff]
